# Association Between Mode of First Delivery and Subsequent Fecundity and Fertility

**DOI:** 10.1001/jamanetworkopen.2020.3076

**Published:** 2020-04-20

**Authors:** Kristen H. Kjerulff, Ian M. Paul, Carol S. Weisman, Marianne M. Hillemeier, Ming Wang, Richard S. Legro, John T. Repke

**Affiliations:** 1 Department of Public Health Sciences, Penn State College of Medicine, Hershey, Pennsylvania; 2 Department of Obstetrics and Gynecology, Penn State College of Medicine, Hershey, Pennsylvania; 3 Department of Pediatrics, Penn State College of Medicine, Hershey, Pennsylvania; 4 Department of Health Policy and Administration, Penn State College of Health & Human Development, University Park, Pennsylvania

## Abstract

**Importance:**

More than 20% of births globally are by cesarean delivery, including more than 30% in the US. Prior studies have reported lower rates of childbearing after cesarean delivery, but it is not clear if this is due to maternal choice or lower conception rates.

**Objective:**

To investigate the association between mode of first delivery and subsequent conceptions and live births.

**Design, Setting, and Participants:**

The First Baby Study was a multicenter prospective cohort study of women aged 18 to 35 years with singleton pregnancies, enrolled and interviewed before first childbirth, who delivered in Pennsylvania from 2009 to 2011 and were followed up for 36 months after delivery (until April 2014). Data analysis for this study took place between May and July 2019 and in January 2020.

**Exposures:**

Mode of first delivery (cesarean or vaginal).

**Main Outcomes and Measures:**

Rates of subsequent conceptions and live births. Discrete-time Cox proportional hazard regression models were used to compare the rate of subsequent conception (vaginal vs cesarean) among those who completed the 36-month survey, accounting for reported months of unprotected intercourse during the follow-up period and adjusting for relevant covariates. A log binomial regression was used to compare the age-adjusted rate of subsequent live birth (vaginal vs cesarean) among those who completed the 36-month survey.

**Results:**

The study population consisted of 2423 women who were retained to the 36-month survey (mean [SD] age at baseline was 27.2 [4.4] years and 712 [29.4%] delivered by cesarean). There were 2046 women who had unprotected intercourse during the follow-up period, 2021 of whom provided data on months of unprotected intercourse. Cesarean delivery was associated with lower rates of conception after unprotected intercourse during the follow-up period (413 of 599 [68.9%]) compared with vaginal delivery (1090 of 1422 [76.7%]) (adjusted hazard ratio, 0.85; 95% CI, 0.74-0.96). Cesarean delivery was also associated with reduced likelihood of a subsequent live birth (305 women [42.8%]) compared with vaginal delivery (857 women [50.1%]), with an age-adjusted risk ratio of 0.83 (95% CI, 0.75-0.92).

**Conclusions and Relevance:**

In the 3 years following first childbirth, women who delivered their first child by cesarean had lower rates of conception after unprotected intercourse, and fewer of these women had a second child than those who delivered vaginally.

## Key Points

**Question:**

Is cesarean delivery at first childbirth associated with a lower rate of subsequent conception compared with vaginal delivery?

**Findings:**

In this cohort study of 2423 women, first childbirth by cesarean delivery was associated with a lower rate of conception after unprotected intercourse when compared with conception rates of women after vaginal delivery. These results remained significant after controlling for relevant covariates.

**Meaning:**

These findings suggest that women who deliver their first child by cesarean delivery may be less likely to conceive a second child in the 3 years following first delivery than women who deliver their first child vaginally.

## Teaser

This cohort study examines the association of first childbirth by cesarean vs vaginal delivery with rates of subsequent conceptions and live births among US women.

## Introduction

The global average annual cesarean delivery rate has more than tripled over the past 25 years, from 6.7% in 1990 to 21.1% in 2015.^1,2^ In the US, nearly a third of deliveries are by cesarean (31.9% in 2018), with rates above 36.0% in several states.^3^ Most studies examining the association between mode of delivery and subsequent childbearing conducted over the past 35 years have reported lower rates of childbearing after cesarean delivery compared with vaginal delivery.^4,5,6^ A meta-analysis^6^ that included nearly 600 000 women reported that women who had cesarean delivery were 9% less likely to have a subsequent pregnancy (age-adjusted risk ratio [RR], 0.91; 95% CI, 0.87-0.95) and 11% less likely to have a subsequent live birth (age-adjusted RR, 0.89; 95% CI, 0.87-0.92). Some have hypothesized that women who deliver by cesarean are less likely to want additional children or more likely to decide to delay subsequent childbearing,^7,8,9,10,11^ while others have hypothesized that women who deliver by cesarean are less likely to conceive subsequently,^12,13,14,15^ either because of the factors that predispose them to need cesarean delivery or because of the cesarean delivery itself.^12,13,14^ Because most of the previous studies of childbearing subsequent to cesarean delivery were retrospective analyses of existing data sets designed for other purposes, such as birth certificate data,^8,16,17,18,19,20^ or were surveys conducted years after the index delivery,^7,21,22^ it is not clear if women who deliver by cesarean are less likely to plan to have additional children and subsequently do not try to conceive, or are simply less likely to conceive.

We designed a large-scale prospective cohort interview study to investigate factors associated with subsequent childbearing following first childbirth. The goals of this study were to compare women who delivered their first child vaginally with those who delivered by cesarean in terms of fecundity (“the biologic capacity for reproduction, irrespective of pregnancy intention”^23^) and fertility (“demonstrated fecundity, measured by live births”^23^).

## Methods

### Study Design

The First Baby Study was a prospective, multicenter, cohort study of nulliparous women who were enrolled during pregnancy and followed up through 36 months post partum. The participants were interviewed during pregnancy (baseline) and then at 1, 6, 12, 18, 24, 30, and 36 months post partum. Key objectives of the postpartum interviews were to measure unprotected intercourse in each of the 36 months of follow-up and resulting conceptions, including those that did not result in a live birth. Women who dropped out of the study before the 36-month survey were missing data concerning unprotected intercourse, as well as conceptions and pregnancy outcomes, for at least 6 of the 36 months of follow-up (and generally more). Therefore, this study was designed and powered to include only those women who were retained to the 36-month data collection stage. Details about sample size and power calculations, participant recruitment, and data collection procedures have been published elsewhere^24^ and are also provided in eMethods 1 and eMethods 2 in the Supplement.

This study was approved by the institutional review board of the Penn State University College of Medicine, as well as the review boards of all hospitals and other institutions involved with participant recruitment. All participants provided written informed consent. The study followed the Strengthening the Reporting of Observational Studies in Epidemiology (STROBE) reporting guidelines.

### Exposure and Outcomes

There were 2 main outcomes measured during the 36 months of follow-up: (1) conception (whether participants conceived after unprotected intercourse) among the subset of women who were retained to the 36-month data collection stage and reported having unprotected intercourse during the follow-up period, for the purpose of comparing women who delivered their first child vaginally with those who delivered by cesarean, and (2) 1 or more live births among all of the women who were retained to the 36-month data collection stage, to compare the women who delivered their first child vaginally with those who delivered by cesarean. The secondary outcomes included number of pregnancies, live births, miscarriages, abortions and stillbirths, months of unprotected intercourse before first conception or resulting in no conception, becoming pregnant for the first time since first delivery at the time of the 36-month survey, and whether women sought fertility advice, testing, or treatment during the follow-up period. There were a small number of women who, for various reasons (such as being in same-sex relationships), used artificial insemination or other types of fertility treatment to attempt conception of 1 or more subsequent children. For these women, we classified the number of months of attempted conception via fertility treatment as number of months of unprotected intercourse.

### Covariates

Preexposure covariates (factors preceding delivery) investigated included demographic and psychosocial factors (stress, depression, and social support), plans to have another child within 3 years, health history (including prepregnancy body mass index, time to conception of first child, and gestational weight gain more than recommended^25^), pregnancy complications, and indications for cesarean delivery. Concurrent exposure covariates included labor and delivery–related complications, as well as in-hospital maternal and newborn morbidities. Additional details describing the assessment of all outcomes and covariates are provided in eMethods 3 in the Supplement.

### Statistical Analysis

Statistical analyses were performed using SPSS version 24.0 (IBM/SPSS Inc) and SAS version 9.4 (SAS Institute Inc). The statistical analyses for this article were performed between May and July 2019 and in January 2020. This study was designed and powered to compare childbearing subsequent to 2 modes of delivery, vaginal vs cesarean, as have most previous studies of mode of delivery in relation to subsequent childbearing.^4,5,6^ However, we compared spontaneous with instrumental vaginal delivery and planned cesarean with unplanned cesarean for our 2 study outcomes to determine whether there were mode of delivery subgroup differences that we would need to adjust for. The 2 vaginal delivery modes (spontaneous and instrumental) were not significantly different at the study’s threshold for statistical significance (2-tailed *P* < .05) on the study outcomes, nor were the 2 cesarean delivery modes (planned and unplanned) (eTable 1 in the Supplement).

We adjusted for clustering within delivery hospitals.^26,27^ For the outcome of rate of conception after unprotected intercourse, we used discrete-time Cox proportional hazards models^28^ to estimate the hazard ratios (HRs) and 95% CIs among women who had unprotected intercourse during the follow-up period and reported months of unprotected intercourse. We measured the associations between the potential covariates and the exposure variable (mode of delivery), as well as the associations between the potential covariates and the primary outcome variable (conception after unprotected intercourse) via χ^2^ analyses to determine which variables to include in the HR models as confounders. Variables that were associated (at *P* ≤ .10) with both the exposure variable and the outcome variable were included as covariates in the Cox proportional hazard models. The association between mode of first delivery and subsequent conception was modeled in stages as follows: model 1 adjusted for age, model 2 adjusted for age and preexposure covariates, and model 3 adjusted for these factors as well as concurrent-exposure covariates. The time variable in these analyses was months of unprotected intercourse. Because the primary objective of this study was to measure fecundity, women were included in these analyses if they reported having unprotected intercourse and reported the number of months of unprotected intercourse during the follow-up period, regardless of whether they reported that they were trying to conceive or not. We constructed curves showing the crude unadjusted cumulative percentage of women who conceived during the follow-up period in association with the number of months of unprotected intercourse for the women who delivered their first child vaginally and by cesarean.

Log binomial regression models were used to estimate the unadjusted and age-adjusted RRs and 95% CIs of a second live birth by 36 months post partum, comparing vaginal with cesarean delivery among all women who completed the 36-month survey. There were no missing data for the main outcomes of conception and subsequent live births within 36 months among the women who completed the 36-month survey, and little or no missing data for the study covariates (<1%). We compared those who were lost to follow-up with those who were retained in terms of maternal age, race/ethnicity, education, poverty status, marital status, mode of delivery, pregnancy intendedness of the first child, and plans to have another child within 3 years after the birth of the first child, via χ^2^ analyses and logistic regression.

## Results

From the enrolled sample of 3006 women, 2423 women (80.1%) completed the 36-month follow-up (eFigure in the Supplement), of whom 2046 (84.4%) reported having unprotected intercourse during the follow-up period, and 2021 (83.4%) women provided complete data on months of unprotected intercourse. There was no significant difference in attrition by delivery mode (eTable 2 in the Supplement). The factors most strongly associated with loss to follow-up, based on logistic regression, were nonwhite race, having less than a college degree, and being unmarried (eTable 3 in the Supplement).

The characteristics of the 2423 study participants can be seen in Table 1. There were 712 women who delivered their first child by cesarean (29.4%), and the mean (SD) age at baseline was 27.2 (4.4) years. Those who delivered by cesarean were older, more likely to be overweight and obese, shorter, and more likely to have sought fertility advice, testing, or treatment. Women did not differ by mode of delivery in prenatal plans for subsequent childbearing. As shown in Table 2, women did not differ by mode of delivery in whether they planned to have a subsequent child during the coming 3 years as reported at 1 month post partum (1009 women [59%] in the vaginal group vs 408 women [57.3%] in the cesarean group; *P* = .47); whether or not they had unprotected intercourse during the follow-up period (1443 women [84.3%] in the vaginal group vs 603 women [84.7%] of the cesarean group; *P* = .85); the mean (SD) age of the first child when the women first began having unprotected intercourse (13.5 [10.2] months for the vaginal group vs 13.2 [10.1] months for the cesarean group; *P* = .64); or the mean (SD) frequency of unprotected intercourse per month in the months when they were sexually active (5.1 [3.4] times per month for the vaginal group vs 4.8 [3.3] times per month for the cesarean group; *P* = .12).

**Table 1.** Baseline Characteristics of Study Cohort by Mode of First Delivery

Characteristic	No. (%)		*P* value^a^
	Vaginal (n = 1711)	Cesarean (n = 712)	
Maternal age, y			
18-24	372 (21.7)	111 (15.6)	<.001
25-29	741 (43.3)	300 (42.1)	
30-35	598 (35.0)	301 (42.3)	
Race/ethnicity			
White, non-Hispanic	1516 (88.6)	619 (86.9)	.72
Black, non-Hispanic	72 (4.2)	35 (4.9)	
Hispanic	63 (3.7)	30 (4.2)	
Other	60 (3.5)	28 (3.9)	
Education			
High school or less	192 (11.2)	81 (11.4)	.52
Some college or technical school	449 (26.2)	171 (24.0)	
College graduate	1070 (62.5)	460 (64.6)	
Private insurance	1429 (83.5)	600 (84.3)	.67
Poverty level^b^			
Poverty	98 (5.7)	40 (5.6)	.99
Near poverty	149 (8.7)	63 (8.8)	
Not poverty	1459 (85.5)	609 (85.5)	
Married	1333 (77.9)	553 (77.7)	.94
Prepregnancy body mass index^c^			
<18.5	55 (3.2)	13 (1.8)	<.001
18.5-24.9	991 (58.0)	312 (43.9)	
25.0-29.9	371 (21.7)	179 (25.2)	
30.0-34.9	180 (10.5)	98 (13.8)	
35.0-39.9	61 (3.6)	63 (8.9)	
≥40	52 (3.0)	46 (6.5)	
Maternal height, in			
53-62	288 (16.8)	188 (26.4)	<.001
63-65	677 (39.6)	257 (38.6)	
≥66	745 (43.6)	249 (35.0)	
Prior miscarriages	270 (15.8)	125 (17.6)	.28
Prior induced abortions	88 (5.1)	23 (3.2)	.04
Active smoking	133 (7.8)	57 (8.0)	.87
Pregnancy was intended	1235 (72.9)	520 (73.6)	.76
Conceived first child while trying to conceive	1173 (68.6)	494 (69.4)	.70
Time to conception of first child among those who tried to conceive, mo			
1-5	799 (68.1)	314 (63.6)	.08
6-12	223 (19.0)	97 (19.6)	
≥13	151 (12.9)	83 (16.8)	
Fertility advice, testing, or treatment	196 (11.5)	111 (15.6)	.01
Plan to have another baby within 3 y	1079 (63.1)	441 (61.9)	.61

^a^ Calculated using a χ^2^ test.

^b^ Poverty categories based on US Census Bureau: poor, family income less than or equal to 100% of federal poverty level; near poor, family income 101% to 200% of federal poverty level; not poor, family income greater than 200% of federal poverty level.^29^

^c^ Calculated as weight in kilograms divided by height in meters squared.

**Table 2.** Postpartum Plans to Have a Second Child and Unprotected Intercourse During 36-Month Follow-up Period by Mode of First Delivery

Characteristic	Vaginal (n = 1711)	Cesarean (n = 712)	*P* value
Plan to have another baby within 3 y reported at 1 mo post partum, No. (%)	1009 (59.0)	408 (57.3)	.47^a^
Had unprotected intercourse, No. (%)^b^	1443 (84.3)	603 (84.7)	.85^a^
Had unprotected intercourse trying to become pregnant, No. (%)^b^	940 (54.9)	379 (53.2)	.45^a^
Had unprotected intercourse not trying to become pregnant, No. (%)^b^	503 (29.4)	224 (31.5)	.33^a^
Age of first child when mother first had unprotected intercourse among women who had unprotected intercourse, mean (SD), mo^c^	13.5 (10.2)	13.2 (10.1)	.64^d^
Frequency of unprotected intercourse per month among women who had unprotected intercourse, mean (SD)^c^	5.1 (3.4)	4.8 (3.3)	.12^d^

^a^ Calculated using a χ^2^ test.

^b^ Unprotected intercourse before first conception or resulting in no conception during the follow-up period.

^c^ Among the 2021 women who reported having unprotected intercourse before first conception or resulting in no conception and reported months of unprotected intercourse.

^d^ Calculated using a *t* test.

Compared with vaginal births, women whose first delivery was by cesarean were less likely to conceive after unprotected intercourse, whether or not they were trying to conceive (Table 3). Among the 2423 women in the study, 305 (42.8%) whose first delivery was by cesarean had 1 or more live births during the 36-month follow-up period, compared with the 857 women (50.1%) whose first delivery was vaginal. The unadjusted RR was 0.85 (95% CI, 0.77-0.94) and the age-adjusted RR was 0.83 (95% CI, 0.75-0.92). Mode of first delivery was not associated with subsequent miscarriage or abortion, but women who had delivered by cesarean had a higher subsequent stillbirth rate than those who had delivered vaginally (6 of 509 [1.2%] vs 2 of 1393 [0.1%]; *P* = .01).

**Table 3.** Subsequent Fertility Outcomes During 36-Month Follow-up Period by Mode of First Delivery

Outcome	Vaginal (n = 1711)	Cesarean (n = 712)	*P* value
**Main outcomes**			
Conceived after unprotected intercourse, No./total No. (%)^a^	1105/1443 (76.7)	414/603 (68.8)	<.001^b^
Conceived after unprotected intercourse, trying to conceive, No./total No. (%)^a^	830/940 (88.3)	312/379 (82.3)	.006^b^
Conceived after unprotected intercourse, not trying to conceive, No./total No. (%)^a^	275/503 (54.7)	103/224 (46.0)	.04^b^
Conceived at least once among the women who used birth control consistently, No./total No. (%)	12/268 (4.5)	6/109 (5.5)	.79^b^
1 ≥ live births, No. (%)	857 (50.1)	305 (42.8)	.001^b^
**Secondary outcomes**			
No. of pregnancies (%)			.02^b^
0	594 (34.7)	291 (40.9)	
1	887 (51.8)	348 (48.9)	
2	196 (11.5)	61 (8.6)	
≥3	34 (3.0)	12 (1.7)	
Miscarriage rate, No./total No. (%)	222/1393 (15.9)	76/509 (14.9)	.47^b^
Stillbirth rate, No./total No. (%)	2/1393 (0.1)	6/509 (1.2)	.01^b^
Abortion rate, No./total No. (%)	24/1393 (1.7)	5/509 (1.0)	.23^b^
Months of unprotected intercourse before first conception, mean (SD)^c^	6.0 (5.4)	6.6 (5.8)	.09^d^
Months of unprotected intercourse resulting in no conception, mean (SD)^e^	9.0 (9.3)	11.4 (10.5)	.009^d^
More than 12 mo of unprotected intercourse resulting in no conception among women who did not conceive after unprotected intercourse, No./total No. (%)	77/332 (23.2)	68/186 (36.6)	.002^b^
Pregnant for first time since first birth as of 36 mo post partum, No. (%)	128 (0.8)	62 (0.9)	.32^b^
No. of live births (%)			.01^b^
0	854 (49.9)	407 (57.2)	
1	823 (48.1)	294 (41.3)	
2	30 (1.8)	10 (1.4)	
≥3	4 (0.2)	1 (0.1)	
Age of first child in months when mother had second live birth, mean (SD)	25.5 (5.9)	25.3 (6.1)	.67^d^
Sought fertility counseling, testing, or treatment, No. (%)	114 (6.7)	51 (7.2)	.66^b^

^a^ Unprotected intercourse before first conception or resulting in no conception.

^b^ Calculated using a χ^2^ test.

^c^ Among the 1503 women who had unprotected intercourse before first conception and reported months of unprotected intercourse.

^d^ Calculated using a *t* test.

^e^ Among the 518 women who had unprotected intercourse resulting in no conception and reported months of unprotected intercourse.

As can be seen in eTables 4 and 5 in the Supplement, the preexposure and concurrent-exposure covariates that were significantly associated with both mode of first delivery and subsequent conception among women who had unprotected intercourse were maternal age, prepregnancy body mass index, time to conception of the first child, gestational weight gain (more than recommended by Institute of Medicine guidelines^25^), prior induced abortions, diabetes, hypertension, soft-tissue disorders, hospitalization during pregnancy, dystocia, failed induction, and an Apgar score below 9/10 at 5 minutes.

Among the 2043 women who reported having unprotected intercourse, 25 reported that they had unprotected intercourse but did not report the months of unprotected intercourse, leaving 2021 women for the analyses involving months of unprotected intercourse, including the discrete-time HR models. Among these women, 599 (29.6%) had delivered their first child by cesarean. Among those who had delivered vaginally, 1090 (76.7%) conceived after unprotected intercourse, while 413 (68.9%) of those who had delivered by cesarean conceived after unprotected intercourse (*P* < .001). As shown in the Figure, women who delivered by cesarean were less likely to conceive than women who delivered vaginally at each value of number of months of unprotected intercourse. Cesarean delivery was associated with a significantly lower rate of conception after unprotected intercourse (age-adjusted HR, 0.77; 95% CI, 0.69-0.87), as shown in Table 4. This association remained significant after adjustment for all covariates (HR, 0.85; 95% CI, 0.74-0.96). In the final model, the following variables were associated with lower rates of conception: cesarean delivery, higher prepregnancy body mass index, taking more than a year to conceive the first child, failed induction at first childbirth, and prior abortions.

**Figure.**
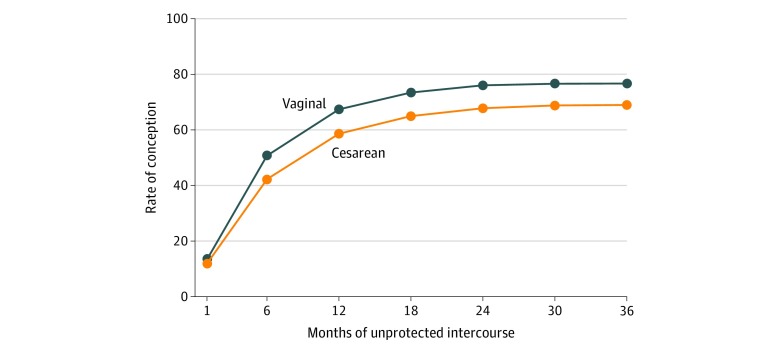
Cumulative Percentages of Women Who Conceived Among 2021 Women Who Reported Months of Unprotected Intercourse

**Table 4.** Rate of Subsequent Conception Among Women Who Had Unprotected Intercourse by Mode of First Delivery, Controlling for Covariates^a^

Characteristic	HR (95% CI)		
	Model 1	Model 2	Model 3
Mode of first delivery (cesarean vs vaginal)	0.77 (0.68-0.87)	0.83 (0.73-0.94)	0.85 (0.74-0.96)
Maternal age, y			
18-24	1 [Reference]	1 [Reference]	1 [Reference]
25-29	1.08 (0.91-1.27)	0.99 (0.83-1.19)	0.99 (0.83-1.19)
30-35	0.93 (0.78-1.10)	0.89 (0.74-1.08)	0.89 (0.74-1.08)
Prepregnancy body mass index^b^			
<18.5		1 [Reference]	1 [Reference]
18.5-24.9		0.72 (0.55-0.96)	0.73 (0.55-0.97)
25.0-29.9		0.71 (0.53-0.96)	0.72 (0.53-0.98)
30.0-34.9		0.72 (0.51-1.00)	0.72 (0.52-1.01)
35.0-39.9		0.58 (0.40-0.83)	0.58 (0.40-0.84)
≥40		0.53 (0.35-0.80)	0.54 (0.35-0.83)
Time to conception of first child in 4 categories			
Conceived first child not trying to conceive		1.44 (1.17-1.77)	1.45 (1.17-1.78)
1-5 mo		2.11 (1.77-2.53)	2.13 (1.78-2.55)
6-12 mo		1.58 (1.29-1.92)	1.59 (1.30-1.94)
≥13 mo		1 [Reference]	1 [Reference]
Gestational weight gain more than recommended^c^		0.92 (0.82-1.04)	0.93 (0.82-1.04)
Prior induced abortions		0.75 (0.57-0.99)	0.75 (0.57-0.99)
Chronic and gestational hypertension or preeclampsia		0.94 (0.79-1.13)	0.96 (0.80-1.15)
Chronic and gestational diabetes or abnormal glucose tolerance		0.85 (0.68-1.07)	0.86 (0.68-1.07)
Soft-tissue disorders		0.98 (0.70-1.36)	0.98 (0.71-1.36)
Hospitalized during pregnancy		1.00 (0.86-1.17)	1.01 (0.86-1.18)
Dystocia			0.98 (0.85-1.12)
Failed induction			0.58 (0.34-0.99)
5-min Apgar <9/10			0.87 (0.73-1.02)

Abbreviation: HR, hazard ratio.

^a^ Among the 2021 women who reported having unprotected intercourse before first conception or resulting in no conception and reported months of unprotected intercourse.

^b^ Calculated as weight in kilograms divided by height in meters squared.

^c^ Gained more than recommended during pregnancy according to Institute of Medicine guidelines.^25^

## Discussion

In this large-scale prospective cohort study of women interviewed before first childbirth and followed for 36 months post partum, we found that cesarean delivery was associated with lower rates of conception after unprotected intercourse during the 36-month follow-up period, and with less likelihood of having a subsequent child than women who had delivered vaginally, even though women who delivered by cesarean were just as likely to plan to have a subsequent child within 3 years after first childbirth, to have unprotected intercourse in the 36 months following first delivery, and to begin having unprotected intercourse at an average of 13 months after first delivery. Although we found that first delivery by cesarean was associated with higher likelihood of subsequent stillbirth compared with vaginal delivery, the number of stillbirths was too small to account for the mode of delivery difference in rate of subsequent live births. These results are in agreement with several previous studies that reported higher stillbirth rates after cesarean delivery.^30,31,32^

While prepregnancy body mass index and time to conception before first childbirth were significant confounding variables in the multivariable models, even after we controlled for these and the other covariates, mode of delivery remained significantly associated with fecundity. In addition, we found this mode of delivery difference in subsequent conception rates even among the women who were having unprotected intercourse while not trying to conceive. However, it is likely that there are other factors involved with fecundity following cesarean delivery that we did not study. In recent years it has been reported that some women develop a defect at the site of the cesarean incision (referred to as *isthmocele* or *niche*) that may increase risk of subsequent infertility.^33^ Such cesarean scar defects have been observed in 61% of women after a single cesarean delivery, based on transvaginal ultrasonographic examination.^34^ It has been reported that the implantation rate and pregnancy rate after in vitro fertilization are significantly lower among women with prior cesarean delivery than those with prior vaginal delivery.^35^

The results of this study are consistent with previous studies, which have reported lower rates of childbearing after cesarean compared with vaginal delivery.^5,6,18^ However, there was 1 previous study of a cohort of primiparous Danish women who were trying to conceive that reported no difference in rate of conception when comparing women who had delivered by unplanned cesarean (112 women) with those who had spontaneous vaginal delivery (585 women), although in that study women who had attempted pregnancy for more than 6 menstrual cycles were excluded.^36^

To our knowledge, this is the first prospective study beginning before first childbirth that measured unprotected intercourse in each month over the course of 36 months after first childbirth, to test whether women who delivered by cesarean were less likely to try to conceive subsequently than women who delivered vaginally, as has been hypothesized, or less likely to conceive after unprotected intercourse. The prospective design of this study was an important strength because we were able to measure women’s plans for subsequent childbearing before and after first childbirth, as well as unprotected intercourse in each month subsequent to first delivery among women who were trying to conceive and those who were not. Additional strengths of this study included a large sample size with adequate power to reject the null hypothesis, and a high retention rate.

### Limitations

This study has several limitations. First, the participants in this study were from 1 state in 1 country, which limits the generalizability of the findings. Second, the 3006 women who were enrolled were of higher socioeconomic status than in the general population of women at first childbirth in the state,^24^ and the 2423 women who were retained to the final survey were of higher socioeconomic status than the 583 women who were lost to follow-up. Bias caused by self-selection and attrition limit the internal validity of an etiologic study if associated with both exposure and outcome.^37^ However, neither self-selection into the study nor attrition was associated with the exposure variable, mode of delivery. Third, the study outcomes of birth control use in each month, unprotected intercourse, pregnancy, stillbirth, miscarriage, and abortion were self-reported, and therefore subject to recall bias and imprecision in the assessment of these factors. Fourth, we only followed women for a period of 3 years after first childbirth. Therefore, we cannot speak to the likelihood that the women in our study who delivered by cesarean would eventually catch up to the women who delivered vaginally in terms of fecundity and fertility. However, long-term studies of mode of first delivery in association with subsequent childbearing have reported lower rates of subsequent live births among women who delivered by cesarean, even after 10 or more years of follow-up.^5,6^

## Conclusions

Our data suggest that first delivery by cesarean is associated with a lower rate of conception during the first 36 months after first childbirth, which is only partially explained by confounding variables. As the global cesarean delivery rate continues to rise, even a modest level of impairment in women’s ability to conceive and bear children after cesarean delivery has the potential to affect the childbearing patterns of many families, particularly in countries with high cesarean delivery rates. Further large-scale prospective studies are needed that measure unprotected intercourse on a month-by-month basis after first childbirth, to see if our findings can be replicated in different populations and to investigate the extent of specific pathologies following cesarean delivery that could explain lower subsequent conception rates among women who have had a previous cesarean delivery.

## Notes

Supplement. **eMethods 1.** Sample Size and Power Calculations **eMethods 2.** Ethical Approval, Inclusion and Exclusion Criteria, and Data Collection Procedures **eMethods 3.** Description of Measurement of Study Variables **eReferences.** **eFigure.** Flow of Participants From Enrollment to the Analytic Cohort **eTable 1.** Comparison of Two Vaginal and Two Cesarean Modes of Delivery on Main Outcomes (Conception and Live Birth) **eTable 2.** Comparison of Women Who Were Lost to Follow-Up to Women Who Were Retained to the 36-Month Survey **eTable 3.** Association of Baseline Characteristics and Loss to Follow-Up, Multivariable Analysis **eTable 4.** Pre-exposure Covariates by Mode of First Delivery and Subsequent Conception Among Women Who Had Unprotected Intercourse Before First Conception or Resulting in No Conception **eTable 5.** Concurrent-exposure Covariates by Mode of First Delivery and Subsequent Conception Among Women Who Had Unprotected Intercourse Before First Conception or Resulting in No Conception
