# Supplementary material for: Association Between Mode of First Delivery and Subsequent Fecundity and Fertility
Source: JAMA Netw Open. 2020 Apr 20;3(4):e203076. doi: 10.1001/jamanetworkopen.2020.3076 (PMC7171551; doi:10.1001/jamanetworkopen.2020.3076)
Supplement: Supplement. — eMethods 1. Sample Size and Power Calculations eMethods 2. Ethical Approval, Inclusion and Exclusion Criteria, and Data Collection Procedures eMethods 3. Description of Measurement of Study Variables eReferences. eFigure. Flow of Participants From Enrollment to the Analytic Cohort eTable 1. Comparison of Two Vaginal and Two Cesarean Modes of Delivery on Main Outcomes (Conception and Live Birth) eTable 2. Comparison of Women Who Were Lost to Follow-Up to Women Who Were Retained to the 36-Month Survey eTable 3. Association of Baseline Characteristics and Loss to Follow-Up, Multivariable Analysis eTable 4. Pre-exposure Covariates by Mode of First Delivery and Subsequent Conception Among Women Who Had Unprotected Intercourse Before First Conception or Resulting in No Conception eTable 5. Concurrent-exposure Covariates by Mode of First Delivery and Subsequent Conception Among Women Who Had Unprotected Intercourse Before First Conception or Resulting in No Conception [file jamanetwopen-3-e203076-s001.pdf]

Supplementary Online Content

Kjerulff KH, Paul IM, Weisman CS, et al. Association between mode of first delivery and subsequent fecundity and fertility. *JAMA Netw Open*. 2020;3(4):e203076. doi:10.1001/jamanetworkopen.2020.3076

eMethods 1. Sample Size and Power Calculations

eMethods 2. Ethical Approval, Inclusion and Exclusion Criteria, and Data Collection Procedures

eMethods 3. Description of Measurement of Study Variables

eReferences.

eFigure. Flow of Participants From Enrollment to the Analytic Cohort

eTable 1. Comparison of Two Vaginal and Two Cesarean Modes of Delivery on Main Outcomes (Conception and Live Birth)

eTable 2. Comparison of Women Who Were Lost to Follow-Up to Women Who Were Retained to the 36-Month Survey

eTable 3. Association of Baseline Characteristics and Loss to Follow-Up, Multivariable Analysis

eTable 4. Pre-exposure Covariates by Mode of First Delivery and Subsequent Conception Among Women Who Had Unprotected Intercourse Before First Conception or Resulting in No Conception

eTable 5. Concurrent-exposure Covariates by Mode of First Delivery and Subsequent Conception Among Women Who Had Unprotected Intercourse Before First Conception or Resulting in No Conception

This supplementary material has been provided by the authors to give readers additional information about their work.

© 2020 Kjerulff et al. *JAMA Netw Open*.

eMethods 1. Sample Size and Power Calculations

Based on analyses of birth certificate records of first births at gestational ages of 34 weeks or later among women aged 18 to 35 years old in Pennsylvania in 2000 matched to subsequent births to the end of 2004, we found that women whose first delivery was vaginal were more likely to have a subsequent delivery within 3 years (41.7%) than those whose first delivery was by cesarean (36.2%), yielding an age-adjusted odds ratio (OR) of 1.29 (95% CI, 1.28-1.31), and an effect size of 0.12 (small effect size). With a significance level of .05 (two-tailed) and a power of .80, the resulting required sample size for this study was 2404. Estimating an attrition rate of 20% over the course of the 36-month follow-up period yielded a minimum enrolled sample size of 3000 study participants.

© 2020 Kjerulff et al. *JAMA Netw Open*.

eMethods 2. Ethical Approval, Inclusion and Exclusion Criteria, and Data Collection Procedures

Ethical approval

The study was approved by the institutional review board (IRB) of the Penn State University College of Medicine, as well as the IRBs of all hospitals and other institutions involved with participant recruitment. All participants provided signed informed consent.

Participants

English and Spanish-speaking women with singleton pregnancies were recruited from prenatal hospital tours, hospital intranet postings, low-income clinics, newspaper ads, childbirth education classes and targeted mailings throughout Pennsylvania. Participants were limited to women aged 18 to 35 years at the time of study enrollment because women beginning childbearing in their later 30's are both more likely to deliver by cesarean and less likely to bear one or more subsequent children.[18] In addition, women were excluded if they delivered before 34 weeks gestation because women who deliver their first child before 34 weeks gestation are less likely to have a subsequent child.[18] Other exclusion criteria included a prior pregnancy of 20 weeks gestation or longer, surrogate pregnancy, plans to have the child adopted, and plans to have a tubal ligation during delivery. The 3006 participants in the FBS were significantly more likely to be white, married, over the age of 29, have private insurance and a college degree than women aged 18 to 35 years at first childbirth in the state of Pennsylvania as a whole, but were not significantly different in mode of delivery.[24] There were 68 women who were lost to follow-up after the baseline interview and 6 women who experienced stillbirth. These 74 women were replaced until we reached our target number of 3000 women who completed both the baseline and 1-month postpartum interviews. We over-enrolled slightly to achieve a sample size of 3006 women who completed both the baseline and 1-month postpartum interviews. Participants delivered at 76 hospitals in Pennsylvania and 2 participants delivered at hospitals in other states.

Data Collection

The baseline interviews were conducted at 30 weeks gestation or later, at a mean (standard deviation (SD)) gestational age of 35.2 (1.6) weeks. Hospital discharge data and birth certificate data for the first childbirth were obtained, as well as birth certificate data for subsequent births that occurred during the 36-month follow-up period. The Pennsylvania Health Care Cost Containment Council (PHC4) used a multi-step process to link the hospital discharge data and birth certificate data to the interview data based on identifiers provided by the study participants, including the mother's first and last name, date of birth, date of delivery, hospital and social security number.

© 2020 Kjerulff et al. *JAMA Netw Open*.

eMethods 3. Assessment of Exposure, Outcomes, and Covariates

Exposure (Mode of Delivery at First Childbirth)

Mode of delivery: In the 1-month interview women were asked "Did you have your baby vaginally or by cesarean section?" Maternal self-report of mode of delivery matched 100%with the diagnosis-related group (DRG) code recorded in the hospital discharge data for mode of delivery.

Planned versus unplanned cesarean: Women who reported that they delivered by cesarean section were then asked "Did you have a planned cesarean section that was scheduled to occur before you went into labor, that is, before regular contractions began, or did you have an unplanned cesarean delivery?" In addition, women were queried extensively about labor, including such questions as: "Were you in labor when you arrived at the hospital?", ""Did a doctor or nurse try to cause your labor to begin by the use of drugs or some other technique? This is often called trying to induce labor." How long had you been in labor when you arrived at the hospital?", "At some point did your contractions become regular and 5 minutes or less apart?, "When you were in labor how painful was it for you during your contractions, before you received any type of pain medication?", and "As best as you can remember, about how many hours was it from the time when you first had regular contractions until you delivered your baby?". Among the 2423 women in this study, there were 127 women who reported that they delivered by planned cesarean delivery. For all 127 women their answers to the questions about labor supported their report that they had cesarean delivery before onset of labor. However, among the 585 women who reported that they had unplanned cesarean, we found 8 women who reported that they were not in labor at the time of hospital admission, were not induced, reported no hours in labor and no pain resulting from labor contractions. In addition, none of the indications for cesarean delivery for these 8 women were labor-related. Therefore we classified these women as having had planned cesarean delivery, for a total of 135 women who had planned cesarean delivery and 577 women who had unplanned cesarean.

Instrumental vaginal: Women were asked "During your labor and birth, did someone "Use forceps to help get the baby out?" and "During your labor and birth, did someone us a vacuum extractor to help get the baby out?" There were 213 women who reported instrumental delivery and an additional 4 women who did not report instrumental vaginal delivery, but instrumental vaginal delivery was reported in the birth certificate data, for a total of 217 women who were classified as having instrumental delivery.

Spontaneous vaginal: Women who reported that they delivered vaginally and were not found to have delivered instrumentally were classified as having had spontaneous vaginal delivery.

Outcome Variables (Measured During the 36-Month Follow-up Period)

Conceived: At the 6, 12, 18, 24, 30 and 36-month data collection stages women were asked "Are you pregnant now?", "How many times have you been pregnant since the previous interview?" and for each pregnancy since the previous interview "How did your first (second, third, etc) pregnancy end"? For each pregnancy women were asked how they knew for sure that they were pregnant, with response options of "Home pregnancy test", "Doctor visit" and "Other". If they answered "Other" they were asked to specify what the other way was. In the 36-month survey we included a section entitled "Pregnancy history recap". In that section women were asked "Now I would like to review your pregnancy history since the birth of your first baby, 3 years ago, just to make sure we have gotten it right. Some of the questions will be the same as we asked before, except that this time we are asking about the entire past 3 years. Since the birth of your first baby about 3 years ago, have you had any pregnancies (including a current pregnancy)?" If they answered yes they were then asked: "How many times have you been pregnant total since the birth of your first child?" If women reported one or more pregnancies at the 6, 12, 18, 24, 30 or 36-month surveys that occurred by the end of the 36th month, or in response to the pregnancy history recap questions, they were classified as having conceived.

© 2020 Kjerulff et al. *JAMA Netw Open*.

Number of conceptions: We counted the number of times that women reported a pregnancy at the 6, 12, 18, 24, 30 and 36-month surveys or in the pregnancy history recap questions that had occurred by the end of the 36th month. Each pregnancy was counted only once.

Pregnant for first time since birth of first child as of 36 months postpartum: Women who answered yes to the question “Are you pregnant now?” asked at the 36-month survey and had not reported any pregnancies in any of the previous surveys or the recap questions were classified as being pregnant for the first time as of the end of the 36th month after the birth of the first child.

Unprotected intercourse before first conception or resulting in no conception: At the 6, 12, 18, 24, 30 and 36-month surveys women were asked about sexual relations. The interviewer said “The next section concerns sexual relations. This is an important part of the study because researchers need to know how the health care that a woman receives at delivery and afterward affects sexual functioning and subsequent fertility.” “Since your last interview have you had sexual intercourse? If they answered “yes” they were then asked: “In each month since we last interviewed you, can you tell me approximately how many times you had intercourse? Let's begin with <M1>”. “Can you tell me how many times you had intercourse in <M1>?” “Can you tell me were there any times when you had sexual intercourse in <M1> without using any type of birth control or protection?” “How many times did you have sexual intercourse in <M1> without using any type of birth control or protection?” “What was the main form of birth control that you used in <M1>?” These questions were then repeated, covering each of the previous 6 months. At each interview women were asked if they were pregnant. If they answered yes they were asked “Were you using any type of birth control such as condoms, withdrawal, or birth control pills at the time your baby was conceived?” In the pregnancy history recap section of the 36-month survey women were asked: “Sometimes women have unprotected intercourse, that is, intercourse without using any type of birth control, even though they are not trying to become pregnant and are not already pregnant. Did you have unprotected intercourse when you were not really trying to become pregnant at any time since the birth of your first baby?” “Did any of the pregnancies that you mentioned above result from unprotected intercourse, even though you were not trying to become pregnant? How many of your pregnancies resulted from unprotected intercourse, even though you were not trying to become pregnant?” Women were considered to have had unprotected intercourse if they reported having unprotected intercourse in one or more months before first conception or resulting in no conception, or if they reported that they were not using birth control at the time of first conception, or if they reported having sexual relations in the months before first conception and reported that the main form of birth control they used was no birth control, or if they reported that they had unprotected intercourse before first conception or resulting in no conception in the recap questions. Women were considered to have had unprotected intercourse resulting in no conception if they reported having unprotected intercourse in one or more of the 36 months of follow-up and reported no conceptions. If they reported only one month of unprotected intercourse we did not count it if it occurred in months 35 or 36 because it would have been too soon to determine if they did not conceive as of the 36-month survey. Some women reported having unprotected intercourse in one or more months before first conception for as many as 10 months, but reported that at the time they conceived they were using birth control. These women were classified as having conceived after unprotected intercourse.

Months of unprotected intercourse: We counted the number of months of unprotected intercourse women reported in the previous 6 months at the 6, 12, 18, 24, 30 and 36-month surveys. Some women reported no months of unprotected intercourse in the 6 to 36-month surveys, but reported 1 or more months of unprotected intercourse before first conception or resulting in no conception in the 36-month pregnancy history recap questions. In that case we used the number of months of unprotected intercourse reported in the recap questions. A small number of women (n = 25) reported having unprotected intercourse before first conception or resulting in no conception but did not report months of unprotected intercourse in the interviews or in the recap questions.

Used birth control consistently in months before first conception or no conception: Women who reported using birth control in each month before first conception or across all 36 months if no conception occurred, and reported

© 2020 Kjerulff et al. *JAMA Netw Open*.

never having unprotected intercourse in any of the months before first conception or no conception, in the 6 to 36-month survey questions and the recap questions, were classified as using birth control consistently. We counted any type of method to prevent pregnancy as using birth control, including withdrawal and the rhythm method. There were 18 women who reported that they conceived their first pregnancy during the follow-up period while using birth control and had also reported no months of unprotected intercourse before that conception.

Trying to become pregnant: In the pregnancy history recap questions in the 36-month survey women were asked "For the first pregnancy after the birth of your first baby, were you trying to become pregnant when the baby was conceived?" Women who answered "yes" to this question were classified as trying to conceive their first pregnancy after the birth of the first child. Women were also asked "Have you tried to become pregnant at any time since the birth of your first child?" Women who had not conceived and answered yes to this question were classified as trying to conceive as well.

Number of miscarriages, stillbirths and abortions: Women were asked to report how each pregnancy they had reported since the previous interview ended, at the 6, 12, 18, 24, 30 and 36-month interviews, including the response options of miscarriage, stillbirth and abortion. In addition, women were asked to report how each pregnancy ended in the 36-month recap questions, in case any pregnancies were missed in the interviews.

One or more live births: In the 12, 18, 24, 30 and 36-month surveys women were asked detailed questions about each live birth that had occurred since the previous interview, including the date of birth, hospital, gender, gestational age, the labor and delivery process and the health of the new baby. In addition, women were asked about all pregnancies and the outcome of all pregnancies reported in the pregnancy history recap section of the 36-month survey. Women were classified as having had a live birth if they reported having a live birth in the interviews or the recap questions, as of the end of the 36th month. Birth certificate data were obtained for live births that occurred during the 36 months of follow-up, unless the women had moved out of state.

Number of live births: The number of live births reported in the interviews and recap questions were counted to obtain a total number of live births which occurred over the course of the 36-month follow-up period, as of the end of the 36th month.

Fertility counseling, testing or treatment: Women who reported being pregnant at any of the interviews were asked "Did you or your partner use any type of fertility advice, testing or treatment before you became pregnant?" In addition, at the 36-month survey women were asked if they had sought fertility advice, testing or treatment at any time since the birth of their first child. Women who reported having sought fertility advice, testing or treatment in any of the interviews were classified as having sought fertility advice, testing or treatment.

Pre-Exposure Covariates

Age: As part of the screening process women were asked their age. Age was verified in relation to the age reported in the birth certificate data.

Race/Ethnicity**:** In the baseline interview women were asked "Do you consider yourself Hispanic or Latina?" and "Do you consider yourself primarily white or Caucasian, Black or African American, Asian, Native Hawaiian or Pacific Islander, American Indian or Alaskan Native, or something else?**"** If women did not answer one or both of these questions we obtained race/ethnicity information from the birth certificate data.

Education: In the baseline interview women were asked **"**What is the highest level of schooling you completed?" If they did not answer this question we obtained this information from the birth certificate data.

Insurance coverage at delivery: This information was obtained from the hospital discharge data. There were 12 women who were initially classified as self-pay in the discharge data. However, 11 of these 12 women reported at the 6 or 12-month survey that their delivery bills had been covered by Medicaid and were reclassified as insured by public insurance.

Poverty level: The US Census Bureau defines the official poverty level based on household income levels for each combination of the total number of people living in the household and the number of these people aged $\geq$ 18 years.[1] Income levels are updated annually for inflation. We used the official U.S. Census Bureau levels for poverty and categories of income related to poverty for 2009, 2010 and 2011, depending on the year the participant delivered her first child. Categories were based on the ratio of income to the poverty level. Participants were categorized as living in poverty if their family income was $\leq$100% of the federal poverty level (FPL), near poor if their family income was 101-200% of the FPL and not poor if their family income was above 200% of the FPL.[1]

Marital status: In the baseline interview women were asked "Are you married and living with your husband, not married but living with a partner, widowed, divorced, separated or never been married?"

Pre-pregnancy BMI: In the baseline interview women were asked "How tall are you in stocking feet?" and "How much did you weigh right before you became pregnant?" If women answered "don't know" or refused to answer the later question, we obtained pre-pregnancy weight from the birth certificate data. Self-report of height and pre-pregnancy weight were compared to the values reported in the birth certificate data. Where there were discrepancies between self-report and birth certificate data we judged which value made most sense in light of the current weight women reported at the time of the baseline interview, weight reported at the time of delivery in the birth certificate data, and current weight reported at the time of the 1-month interview. Pre-pregnancy BMI was categorized as underweight (< 18.5 kg/m^2), normal weight (18.5-24.9 kg/m^2), overweight (25.0-29.9 kg/m^2), class 1 obese (30 to < 35 kg/m^2), class 2 obese (35-<40 kg/m^2) and class 3 obese $\geq$ 40.0 kg/m^2).

Maternal height: In the baseline interview women were asked "How tall are you in stocking feet?"

Prior Miscarriages and Abortions: In the baseline interview women were asked "How many times have you been pregnant in your life counting your current pregnancy?" and how each prior pregnancy had ended, including miscarriages and abortions. Because women who had a pregnancy of 20 weeks or longer were excluded, none of the study participants would have had a prior stillbirth.

Smoker: In the baseline interview women were asked "During your current pregnancy have you smoked cigarettes every day, some days or not at all?" Women who answered "every day" or "some days" were classified as being a smoker.

Pregnancy intendedness: In the baseline interview women were asked "Thinking back to just before you got pregnant this time, how did you feel about becoming pregnant?" with response options of "You wanted to be pregnant sooner", "You wanted to be pregnant later", "you wanted to be pregnant then" and "You didn't want to be pregnant then or at any time in the future".[2] If women answered that they wanted to be pregnant later or never, they were classified as having an unintended pregnancy.

Conceived 1st child while trying to conceive: In the baseline interview women were asked "At the time you conceived were you trying to conceive?"

Time to conception of 1st child among those trying to conceive: Women who answered "yes" to the above question were then asked "How long did it take for you to become pregnant from the time that you began actively trying to conceive?" This variable was highly skewed, so it was organized into three categories, 1-5 months, 6-12 months and 13 or more months.

Time to conception of 1st child in four categories: The above two variables were combined into one variable for use in the multivariable models. The four categories were "Conceived 1st child not trying to conceive, 1-5 months, 6-12 months, and 13+ months.

Fertility advice/testing or treatment before first childbirth: In the baseline interview women were asked "Did you or your partner use any type of fertility advice, testing or treatment before you become pregnant?"

Plans to have another baby within 3 years: In the baseline interview women were asked a series of questions about plans for future childbearing, including "Do you have plans as to when you would like to have another baby after

© 2020 Kjerulff et al. *JAMA Netw Open*.

you have this one?” and “If so, when do you plan to have another baby after this one?”, with response options of “Within a year”, “About 2 years”, “About 3 years”, “About 4 years”, “5 or more years”, and “don’t know”. Women who reported that they planned to have another baby within a year, 2 years or 3 years were classified as planning to have another baby within 3 years.

Mode of delivery preference: In the baseline interview women were asked “At this point would you prefer to have your baby by cesarean section or have a vaginal delivery?” Response options were “Vaginal”, “Cesarean” and “No preference”.[3]

Fear of childbirth: In the baseline interview women were administered the FBS-CAS (First Baby Study Childbirth Anticipation Scale),[4] and were asked to indicate the extent to which they had specific feelings about the upcoming delivery, using the rating scale of “Extremely”, “Quite a bit”, “Moderately”, “A Little” and “Not at all”. Women’s ratings of the extent to which they felt nervous, worried, fearful and terrified were summated to create a total score of fear of childbirth. The Cronbach’s alpha for this scale was 0.84. Total scores could range from 4 (no fear) to 20 (high fear). Total scores were classified into three categories: 4-8 (low fear), 9-12 (medium fear), and 13-20 (high fear).

Depression during pregnancy: As part of the baseline interview participants were administered the Edinburgh Depression Scale (EDS).[5] The EDS is a 10-item inventory which asks respondents to report how they have been feeling in the past week, with items such as “I have looked forward with enjoyment to things.” and “I have been so unhappy I have been crying.” Total scores could range from 0 (no depression) to 30 (high depression). The overall Cronbach’s alpha was 0.79. We used the cutoff score of 13 or above as indicative of likely depression.[6]

Social support during pregnancy: Social support was measured using a 5-item shortened version of the Medical Outcomes Study (MOS) Social Support Scale.[7] Total scores could range from 5 to 25, with higher scores indicating higher social support. The Cronbach’s alpha for this scale was 0.88. Total scores were classified into three categories of social support: 5-19 (low), 20-23 (medium), and 24-25 (high).

Stress during pregnancy: Stress was measured using a modified version of the Psychosocial Hassles Scale (PHS).[8,9] In the PHS respondents are asked to rate the degree of stress they have experienced during the pregnancy due to specific factors such as “Money worries like paying bills”. Based on pilot testing of the study questionnaires two of the items in the original version of the instrument exhibited poor corrected item-total correlations. These items were “Sexual, emotional or physical abuse” and “Problems with alcohol or drugs”. Based on focus group studies discussing common problems experienced during pregnancy we changed these items to “Fights with partner” and “Fights with other family members”. These new items worked well and exhibited good corrected item-total correlations. Total scores could range from 12 (no stress) to 48 (high stress). The overall Cronbach’s alpha for the scale was 0.76. Total scores were classified into three categories: 12-16 (low stress), 17-20 (medium stress), and 21-48 (high stress).

Gestational weight gain: In the 1-month postpartum interview women were asked “How much total weight had you gained during this pregnancy?” If they answered “don’t know” we obtained that information from the birth certificate data. Based on the 2009 Institute of Medicine guidelines,[10] which takes into account women’s pre-pregnancy BMI, women were categorized as gaining less than recommended, as recommended, or more than recommended.

Hospitalized during pregnancy: In the baseline interview women were asked “During this pregnancy have you been hospitalized for any reason?” In the 1-month postpartum interview women were asked a series of questions about hospitalizations since the baseline interview but before delivery, including “Were you admitted to the hospital?” and “Considering all hospitalizations, how many days total were you in the hospital in the time period after the first interview, but before you went to the hospital to have your baby?” Women who reported being hospitalized during pregnancy, before or after the baseline interview, were classified as having been hospitalized during pregnancy. This included women who were hospitalized during pregnancy before the onset of labor, such as for preeclampsia, and then delivered during that hospitalization.

© 2020 Kjerulff et al. *JAMA Netw Open*.

The following pre-exposure conditions were measured primarily via the ICD-9 CM codes reported in the hospital discharge data, as described in Korst et al (2014).[11] Women were also classified as having each condition if it was reported in the birth certificate data or by maternal self-report. Following are the ICD-9 CM codes used to identify each condition.

Chronic and gestational hypertension and preeclampsia: ICD-9 codes 642.0-642.9, 401-405.9. In addition, women were classified as having one or more of these conditions if it was reported in the birth certificate data (prepregnancy hypertension, gestational hypertension or preeclampsia, or eclampsia) or by maternal self-report.

Chronic and gestational diabetes and abnormal glucose tolerance: ICD-9 codes 648.0, 648.8, 250-250.9. In addition women were categorized as having one or more of these conditions if pre-pregnancy diabetes or gestational diabetes were reported in the birth certificate data or by maternal self-report.

Antepartum bleeding or placental conditions: ICD-9 codes 641.00-641.92, 656.7-656.9. In addition, women were categorized as having these conditions if they self-reported antepartum bleeding or problems with the placenta as a pregnancy complication or condition leading to hospitalization during pregnancy.

Thyroid disorder: ICD-9 code 648.1.

Hydramnios/Oligohydramnios: ICD-9 codes 657.0-658.0. In addition, women were categorized as having these conditions if they self-reported having too much or too little amniotic fluid as a pregnancy complication or condition leading to hospitalization during pregnancy.

Soft tissue disorders including uterine fibroids and endometriosis: ICD-9 codes 654.0,1,4,5,6,7,9; 218.0, 617.0. In addition, women were categorized as having uterine fibroids or endometriosis if they reported in the baseline interview that they had been told by a doctor or nurse that they had uterine fibroids and/or endometriosis.

Fetal intrauterine growth restriction/slow fetal growth: ICD-9 codes 656.5 and 764.9.

Fetal distress/abnormalities in heart rate or rhythm: ICD-9 codes 656.3, 659.7.

Macrosomia: ICD-9 code 656.6.

Breech: ICD-9 code 652.2. In addition, women were categorized as breech if the fetal presentation reported in the birth certificate data was breech.

Other malpresentation: ICD-9 codes 652.0 (unstable lie), 652.3 (transverse or oblique presentation), 652.4 (face or brow presentation), 652.7 (prolapse arm), 652.8 (other specified malposition or malpresentation), 652.9 (unspecified malpresentation).

Concurrent Exposure Covariates

Gestational age: To calculate gestational age in days we compared the delivery due date women reported in the baseline interview to the delivery date reported in the birth certificate data. This method was in agreement with the obstetrician estimated gestational age (within a week) reported in the birth certificate data for 87% of the births. When it was not in accordance we used the obstetrician estimated gestational age reported in the birth certificate data. We further verified gestational age in relation to the ICD-9 diagnostic codes indicating that the child was born pre-term or post-term. Although we also calculated gestational age based on the last menses date reported in the birth certificate data, this later method was generally not consistent with the other two methods of calculating gestational age.

Newborn birth weight: Newborn birth weight was obtained from the birth certificate data.

Newborn sex: In the 1-month survey women were asked "Did you have a boy or a girl?"

Maternal hospital stay > 5 days: The length of hospital stay for the mother was obtained from the hospital discharge data.

© 2020 Kjerulff et al. *JAMA Netw Open*.

5 minute Apgar < 9: The 5-minute Apgar score was obtained from the birth certificate data.

Neonatal Intensive Care Unit (NICU) admission: Neonatal intensive care unit (NICU) admission was obtained from the birth certificate data.

Neonatal hospital stay > 5 days: The length of hospital stay for the newborn was obtained from the hospital discharge data.

The following concurrent-exposure conditions were measured primarily via the ICD-9 CM codes reported in the hospital discharge data, as described in Korst et al (2014).[11] Women were also classified as having each condition if it was reported in the birth certificate data or by maternal self-report. Following are the ICD-9 CM codes used to identify each condition.

Dystocia: ICD-9 codes 660.0-660.9 (except 660.7) for obstructed labor; 661.0-661.9 (except 661.3) for abnormality of forces of labor; and ICD-9 codes 662.0-662.2 for prolonged labor.

Cephalopelvic disproportion: ICD-9 codes 653.0-653.9.

Failed Induction: ICD-9 codes 659.0-659.1.

Failed vacuum or forceps: ICD-9 code 660.7.

Premature or prolonged rupture of membranes and/or amnionitis: ICD-9 codes of 658.1-658.9.

Umbilical cord complications: ICD-9 codes 663.0-663.9.

Perineal laceration, 3rd or 4th degree: ICD-9 codes 664.2, 664.3. In addition, women were classified as having this condition if third or fourth degree perineal laceration was reported in the birth certificate data.

Cesarean wound complications: ICD-9 code 674.1 (disruption of cesarean wound). In addition, in the 1-month interview women were asked a series of questions about health problems they had experienced since the delivery, including "infection at the site of cesarean incision". Women who reported having had an infection at the site of the cesarean incision were classified as having a cesarean wound complication as well.

Fetal congenital anomalies: Newborns were classified as having a congenital anomaly if one or more of the following conditions were reported in the newborn ICD-9 codes: Anencephalus (740.0,1,2); Spina bifida (741): Certain congenital musculoskeletal deformities (754.00-754.89); Cleft palate and cleft lip (749.00-749.25); Chromosomal anomalies (758.0-758.9); Congenital anomalies of the cardiac septum (745.0-745.9); Congenital anomalies of the eye (743.0-743.9): Congenital anomalies of the respiratory system (748.0-748.9); Congenital anomalies of the urinary system (753.0-753.9); Congenital anomalies of the upper alimentary tract (750.03-750.9); Ichthyosis congenital (757.1); Other congenital anomalies of the digestive system (751.0-751.9); Other congenital anomalies of the heart (746.0-746.9, 747.0-747.4); Other congenital anomalies of the nervous system (742.0-742.5, 742.8-742.9); and Other and unspecified congenital anomalies (759.00-759.90).

© 2020 Kjerulff et al. *JAMA Netw Open*.

eReferences

1. US Census Bureau Poverty. http://www/census.gov. Accessed September 17, 2013.

2. Finer LB, Henshaw SK. Disparities in rates of unintended pregnancy In the United States, 1994 and 2001. *Perspect Sex Reprod Health*. 2006;38(2):90-96. doi:10.1363/3809006

3. Kjerulff KH, Attanasio LB, Edmonds JK, Repke JT. Mode of delivery preference among pregnant nulliparous women. *J Womens Health (Larchmt)*. November 2018. doi:10.1089/jwh.2018.6989

4. Elvander C, Cnattingius S, Kjerulff KH. Birth experience in women with low, intermediate or high levels of fear: Findings from the First Baby Study. *Birth*. 2013;40(4):289-296. doi:10.1111/birt.12065

5. Cox J, Chapman G, Murray D, Jones P. Validation of the Edinburgh Postnatal Depression Scale (EPDS) in non-postnatal women. *J Affect Disord*. 1996;39 (3):185-189. doi:10.1016/0165-0327(96)000008-0

6. Matthey S, Henshaw C, Elliott S, Barnett B. Variability in use of cut-off scores and formats on the Edinburgh Postnatal Depression Scale - Implications for clinical and research practice. *Arch Womens Ment Health*. 2006;9(6):309-315. doi:10.1007/s00737-006-0152-x

7. McCarrier K, Bushnell D, Martin M, Paczkowski R, Nelson D, Buesching D. Validation and psychometric evaluation of a 5-Item measure of perceived social support. *Value Heal*. 2011;14(3):A148. doi:10.1016/j.jval.2011.02.824

8. Misra DP, O'Campo P, Strobino D. Testing a sociomedical model for preterm delivery. *Paediatr Perinat Epidemiol*. 2001;15(2):110-122. doi:10.1046/j.1365-3016.2001.00333.x

9. Phelan AL, DiBenedetto MR, Paul IM, Zhu J, Kjerulff KH. Psychosocial stress during first pregnancy predicts infant health outcomes in the first postnatal year. *Matern Child Health J*. 2015;19(12)2587-2597. doi:10.1007/s10995-015-1777-z

10. IOM. *Weight Gain During Pregnancy: Reexamining the Guidelines*.; 2009. doi:10.1097/00006250-196901000-00025

11. Korst LM, Fridman M, Lu MC, et al. Monitoring childbirth morbidity using hospital discharge data: Further development and application of a composite measure. *Am J Obstet Gynecol*. 2014;211 (3):268.e1-268e16. doi:10.1016/j.ajog..2014.03.011

12. Ware J. *How to Score Version 2 of the SF-12 Health Survey*.; 2002. doi:10.1016/j.jpowsour.2008.01.091

eFigure. Flow of Participants from Enrollment to the Analytic Cohort

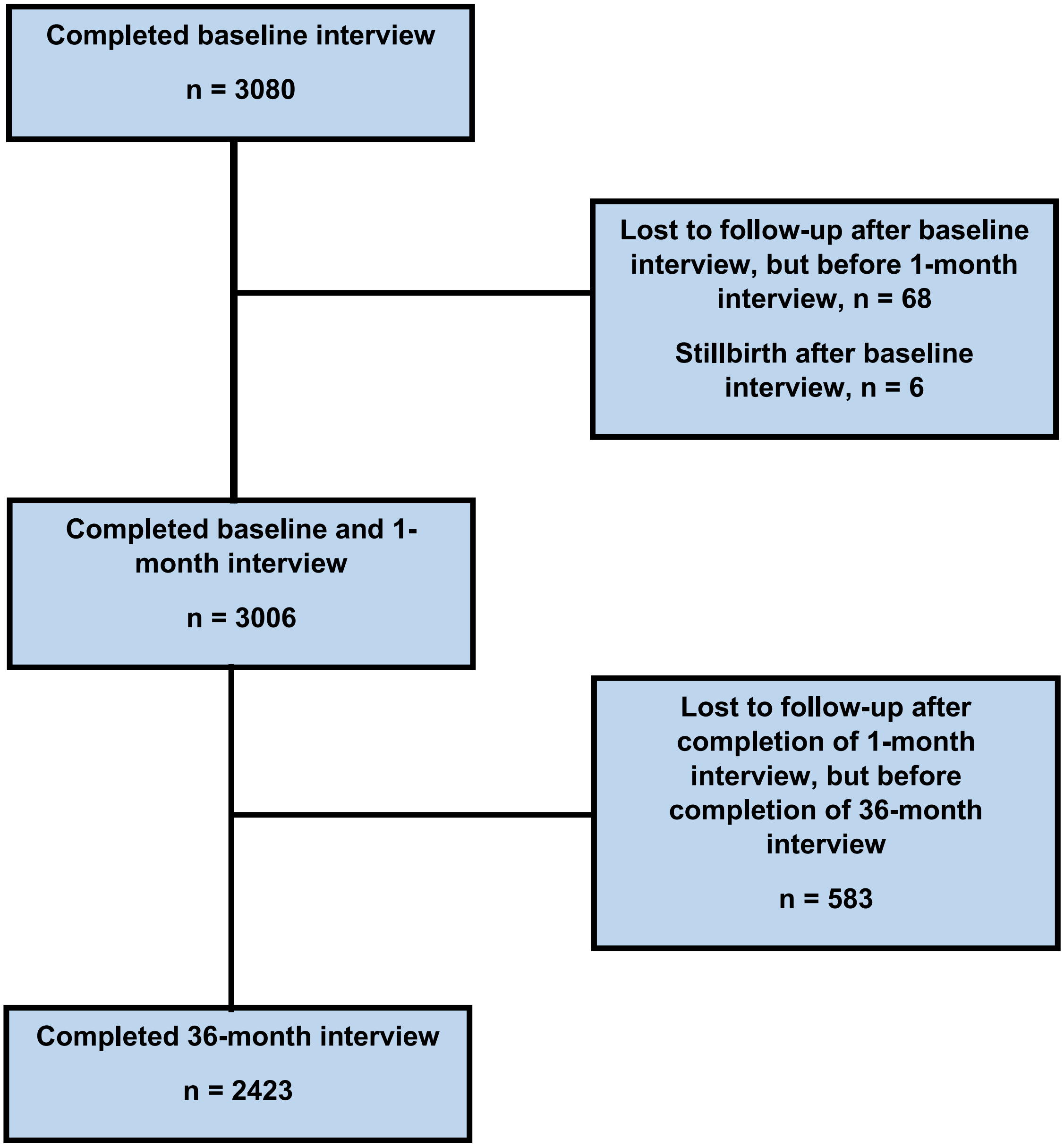

© 2020 Kjerulff et al. *JAMA Netw Open*.

eTable 1. Comparison of Two Vaginal and Two Cesarean Modes of Delivery[a]

	Conceived[b]	*P* Value	Live birth[c]	*P* Value
Vaginal		.07		.77
Spontaneous, No./total No. (%)	962/1269 (75.8)		746/1494 (49.9)	
Instrumental, No./total No. (%)	143/174 (82.2)		111/217 (51.2)	
Cesarean		.38		.70
Planned, No./total No. (%)	77/118 (65.3)		60/135 (44.4)	
Unplanned, No./total No. (%)	338/485 (69.7)		245/577 (42.5)	

[a]All results reported in this table are from χ^2 analyses.

[b]Among women who had unprotected intercourse before first conception or resulting in no conception (n = 2046).

[c]Among women who completed the 36-month survey (n = 2423).

© 2020 Kjerulff et al. *JAMA Netw Open*.

eTable 2. Comparison of Women Who Stayed in the Study (to the 36-Month Survey) to Those Who Were Lost to Follow-Up[a]

Variable	Stayed in study 2423 (80.61%)	Lost to follow-up 583 (19.30%)	*P* Value
Mode of delivery, No. (%)			.10
Vaginal	1711 (70.6)	432 (74.1)	
Cesarean	712 (29.4)	151 (25.9)	
Maternal age, y, No. (%)			< .001
18-24	483 (19.9)	328 (56.3)	
25-29	1041 (43.0)	152 (26.1)	
30-35	899 (37.1)	103 (17.7)	
Race/ethnicity, No. (%)			< .001
White, non-Hispanic	2135 (88.1)	367 (63.1)	
Black, non-Hispanic	107 (4.4)	114 (19.6)	
Hispanic	93 (3.8)	73 (12.5)	
Other	88 (3.6)	28 (4.8)	
Education, No. (%)			<.001
High school degree or less	273 (11.3)	228 (39.1)	
Some college or technical	620 (25.6)	184 (31.6)	
College graduate	1530 (63.1)	171 (29.3)	
Private insurance, No. (%)	2029 (83.7)	283 (48.7)	< .001
Poverty level, No. (%)			
Poverty	138 (5.7)	117 (20.2)	
Near poverty	212 (8.8)	128 (22.1)	
Not poverty	2068 (85.5)	335 (57.8)	
Married, No. (%)	1886 (77.8)	231 (39.6)	<.001
Pregnancy was intended, No. (%)	1755 (73.1)	267 (46.5)	<.001
Plan to have another baby within 3 years reported during pregnancy, No. (%)	1520 (62.7)	228 (39.1)	<.001

[a]All results reported in this table are from χ^2 analyses.

© 2020 Kjerulff et al. *JAMA Netw Open*.

eTable 3. Association between Baseline Characteristics and Loss to Follow-Up, Multivariable Analysis

	Odds Ratio (95% CI)[a]	Wald *P* Value
Vaginal delivery	1.16 (0.92-1.46)	.22
Maternal age, y		
18-24	[Reference]	
25-29	0.65 (0.48-0.87)	.004
30-35	0.62 (0.43-0.88)	.008
Race/ethnicity		
White, non-Hispanic	[Reference]	
Black, non-Hispanic	2.07 (1.47-2.92)	<.001
Hispanic	2.04 (1.40-2.95)	<.001
Other	1.56 (0.96-2.52)	.07
Education		
High school degree or less	[Reference]	
Some college or technical	0.62 (0.47-0.82)	.001
College graduate	0.49 (0.35-0.69)	<.001
Private insurance	0.71 (0.53-0.96)	.03
Poverty level		
Poverty	[Reference]	
Near poverty	0.85 (0.59-1.22)	.38
Not poverty	0.80 (0.56-1.15)	.23
Married	0.62 (0.46-0.84)	.003
Pregnancy was intended	0.89 (0.70-1.14)	.37
Plan to have another baby within 3 years reported during pregnancy	0.76 (0.61-0.95)	.01

[a]Logistic regression, all variables entered into one equation.

© 2020 Kjerulff et al. *JAMA Netw Open*.

eTable 4. Pre-Exposure Covariates by Mode of First Delivery and Subsequent Conception among Women who Had Unprotected Intercourse Before First Conception or Resulting in no Conception[a, b]

	Mode of Delivery			Conceived		
	**Vaginal 1422 (70.4)**	**Cesarean 599 (29.6)**	***P* Value**	**Yes 1503 (74.4)**	**No 518 (25.6)**	***P* Value**
Maternal age, y, No. (%)			.006			<.001
18-24	277 (19.5)	84 (14.0)		236 (15.7)	125 (34.6)	
25-29	629 (44.2)	265 (44.2)		693 (46.1)	201 (38.8)	
30-35	516 (36.3)	250 (41.7)		574 (38.2)	192 (37.1)	
Race/ethnicity, No. (%)			.54			.001
White non-Hispanic	1274 (89.6)	524 (87.5)		1358 (90.4)	440 (84.9)	
Black non-Hispanic	56 (3.9)	26 (4.3)		44 (2.9)	38 (7.3)	
Hispanic	46 (3.2)	25 (4.2)		49 (3.3)	22 (4.2)	
Other	46 (3.2)	24 (4.0)		52 (3.5)	18 (3.5)	
Education, No. (%)			.85			<.001
High school or less	145 (10.2)	65 (10.9)		131 (8.7)	79 (15.3)	
Some college or technical	338 (23.8)	137 (22.9)		310 (20.6)	165 (31.9)	
College graduate	939 (66.0)	397 (66.3)		1062 (70.7)	274 (52.9)	
Private insurance, No. (%)	1208 (85.0)	516 (86.1)	.54	1333 (88.7)	391 (75.5)	<.001
Poverty level, No. (%)[c]			.97			<.001
Poverty	75 (5.3)	30 (5.0)		79 (5.3)	26 (5.0)	
Near poverty	110 (7.8)	47 (7.8)		84 (5.6)	73 (14.1)	
Not poverty	1234 (87.0)	522 (87.1)		1339 (89.1)	417 (80.8)	
Married, No. (%)	1149 (80.8)	482 (80.5)	.85	1304 (86.8)	327 (63.1)	<.001

© 2020 Kjerulff et al. *JAMA Netw Open*.

eTable 4. Pre-Exposure Covariates by Mode of First Delivery and Subsequent Conception among Women who Had Unprotected Intercourse Before First Conception or Resulting in no Conception[a, b] (continued)

	Mode of Delivery			Conceived		
	**Vaginal 1422 (70.4)**	**Cesarean 599 (29.6)**	***P* Value**	**Yes 1503 (74.4)**	**No 518 (25.6)**	***P* Value**
Pre-pregnancy BMI			<.001			<.001
< 18.5	46 (3.2)	11 (1.8)		46 (2.1)	11 (3.1)	
18.5-24.9	837 (58.9)	271 (45.3)		864 (57.5)	244 (47.2)	
25.0-29.9	302 (21.2)	145 (24.2)		325 (21.6)	122 (23.6)	
30-34.9	145 (10.2)	77 (12.9)		155 (10.3)	67 (13.0)	
35-39.9	51 (3.6)	55 (9.2)		68 (4.5)	38 (7.4)	
40.0+	41 (2.9)	39 (6.5)		45 (3.0)	35 (6.8)	
Conceived 1st child while trying to conceive, No. (%)	1016 (71.4)	432 (72.1)	.79	1155 (76.8)	296 (57.1)	<.001
Time to conception of 1st child among those who tried to conceive, No. (%)			.08			<.001
1-5 months	694 (68.3)	273 (63.2)		792 (68.8)	175 (59.1)	
6-12 months	193 (19.0)	86 (19.9)		219 (19.0)	60 (20.3)	
13+ months	129 (12.7)	73 (16.9)		141 (20.6)	61 (20.6)	
Time to conception of 1st child in 4 categories, No. (%)			.153			<.001
Conceived 1st child while not trying to conceive	406 (28.6)	167 (27.9)		351 (23.4)	222 (42.9)	
1-5 months	694 (48.8)	273 (45.6)		792 (52.7)	175 (33.8)	
6-12 months	193 (13.6)	86 (14.4)		219 (14.6)	60 (11.6)	
13+ months	129 (9.1)	73 (12.2)		141 (9.4)	61 (11.8)	

© 2020 Kjerulff et al. *JAMA Netw Open*.

eTable 4. Pre-Exposure Covariates by Mode of First Delivery and Subsequent Conception among Women who Had Unprotected Intercourse Before First Conception or Resulting in no Conception[a, b] (continued)

	Mode of Delivery			Conceived		
	**Vaginal 1422 (70.4)**	**Cesarean 599 (29.6)**	***P* Value**	**Yes 1503 (74.4)**	**No 518 (25.6)**	***P* Value**
Gestational weight gain, No. (%)[e]			<.001			.01
Less than recommended	174 (12.2)	48 (8.0)		162 (10.8)	60 (11.6)	
Recommended	553 (38.9)	166 (27.8)		562 (37.4)	157 (30.3)	
More than recommended	695 (48.9)	383 (64.2)		777 (51.8)	301 (58.1)	
Maternal height, inches, No. (%)			<.001			.35
53-62	233 (16.4)	158 (26.4)		283 (18.8)	108 (20.8)	
63-65	556 (39.1)	224 (37.4)		593 (39.5)	187 (36.1)	
66+	633 (44.5)	217 (36.2)		627 (41.7)	223 (43.1)	
Prior miscarriages, No. (%)	227 (16.0)	108 (18.0)	.27	261 (17.4)	74 (14.3)	.11
Prior induced abortions, No. (%)	69 (4.9)	18 (3.0)	.07	54 (3.6)	33 (6.4)	.01
Smoker, No. (%)	104 (7.3)	40 (6.7)	.64	89 (5.9)	55 (10.6)	.00
Fertility advice, testing or treatment, No. (%)	177 (12.4)	102 (17.0)	.01	209 (13.9)	70 (13.5)	.88
Pregnancy was intended, No. (%)	1068 (75.7)	450 (75.8)	1.00	1207 (81.0)	311 (60.4)	<.001
Edinburgh Depression Score $\geq$ 13, No. (%)	43 (3.0)	23 (3.8)	.34	43 (2.9)	23 (4.4)	.09
MOS Social Support, No. (%)			.24			.02
Low (5-19)	224 (15.8)	103 (17.2)		223 (14.9)	104 (20.1)	
Medium (20-23)	606 (42.7)	271 (45.2)		656 (43.7)	221 (42.7)	
High (24-25)	590 (41.5)	225 (37.6)		622 (41.4)	193 (37.3)	
PHS Stress, No. (%)			.90			<.001
Low (12-16)	514 (36.2)	212 (35.2)		571 (38.0)	155 (30.0)	
Medium (17-20)	558 (39.3)	234 (39.1)		592 (39.4)	200 (38.8)	
High (21-48)	348 (24.5)	152 (25.4)		339 (22.6)	161 (31.2)	
Chronic and gestational hypertension and preeclampsia, No. (%)	167 (11.7)	100 (6.7)	.003	187 (12.4)	80 (15.5)	.08
Chronic and gestational diabetes and abnormal glucose tolerance, No. (%)	87 (6.1)	49 (8.2)	.10	90 (6.0)	46 (8.9)	.03
Antepartum bleeding or placental conditions, No. (%)	139 (9.8)	66 (11.0)	.42	151 (10.0)	54 (10.4)	.80

© 2020 Kjerulff et al. *JAMA Netw Open*.

eTable 4. Pre-Exposure Covariates by Mode of First Delivery and Subsequent Conception among Women who Had Unprotected Intercourse Before First Conception or Resulting in no Conception[a, b] (continued)

	Mode of Delivery			Conceived		
	**Vaginal 1422 (70.4)**	**Cesarean 599 (29.6)**	***P* Value**	**Yes 1503 (74.4)**	**No 518 (25.6)**	***P* Value**
Thyroid disorder, No. (%)	40 (2.9)	30 (5.2)	.02	55 (3.7)	15 (3.0)	.49
Hydramnios/Oligohydramnios, No. (%)	58 (4.1)	37 (6.2)	.05	71 (4.7)	24 (4.6)	1.00
Soft tissue disorders including uterine	28 (2.0)	34 (5.7)	<.001	40 (2.7)	22 (4.2)	.08
Fetal intrauterine growth restriction/slow	47 (3.3)	20 (3.3)	1.00	51 (3.4)	16 (3.1)	.89
Fetal distress/abnormalities in heart rate or	249 (17.5)	194 (32.4)	<.001	339 (22.6)	23.5 (20.1)	.27
Macrosomia, No. (%)	17 (1.2)	54 (9.0)	<.001	53 (3.5)	18 (3.5)	1.00
Breech, No. (%)	8 (0.6)	78 (13.0)	<.001	67 (4.5)	19 (3.7)	.53
Other malpresentation, No. (%)	46 (3.2)	112 (18.7)	<.001	114 (7.6)	44 (8.5)	.51
Hospitalized during pregnancy, No. (%)	219 (15.4)	118 (19.7)	.02	232 (15.4)	105 (20.3)	.01
Mode of delivery preference, No. (%)			<.001			.28
Cesarean	24 (1.7)	33 (5.5)		40 (2.7)	17 (3.3)	
Vaginal	1370 (96.4)	540 (90.2)		1427 (95.0)	483 (93.2)	
No preference	27 (1.9)	26 (4.3)		35 (2.3)	18 (3.5)	
Fear of childbirth, No. (%)			.61			<.001
Low (4-8)	491 (34.5)	194 (32.4)		537 (35.7)	148 (28.6)	
Medium (9-12)	614 (43.2)	263 (43.9)		659 (43.8)	218 (42.1)	
High (13-20)	317 (22.3)	142 (23.7)		307 (20.4)	152 (29.4)	
Plan to have another baby within 3 years, No. (%)	955 (67.2)	387 (64.6)	.28	1083 (72.1)	259 (50.0)	<.001

Abbreviations: BMI, body mass index; MOS, Medical Outcomes Study; PHS, Psychosocial Hassles Scale.

[a]Among women who reported having unprotected intercourse before first conception or resulting in no conception and who reported months of unprotected intercourse (n = 2021).

[b]All results reported in this table are from χ^2 analyses.

[c]Poverty categories based on US Census Bureau: Poor, family income $\leq$ 100% of federal poverty level (FPL); Near poor, family income 101-200% of FPL; Not poor, family income above 200% of FPL.[1c]

[d]Calculated as weight in kilograms divided by height in meters squared.

[e]Based on IOM guidelines[10]

© 2020 Kjerulff et al. *JAMA Netw Open*.

eTable 5. Concurrent-Exposure Covariates by Mode of First Delivery and Subsequent Conception among Women who Had Unprotected Intercourse Before First Conception or Resulting in no Conception[a, b]

	Mode of Delivery			Conceived		
	**Vaginal 1422 (70.4)**	**Cesarean 599 (29.6)**	***P* Value**	**Yes 1503 (74.4)**	**No 518 (25.6)**	***P* Value**
Gestational age, No. (%)			.03			.23
Preterm (34-36 weeks)	55 (3.9)	23 (3.8)		51 (3.4)	27 (5.2)	
Early term (37-38 weeks)	268 (18.8)	111 (18.5)		288 (19.2)	91 (17.6)	
Full term (39-40 weeks)	885 (62.2)	343 (57.3)		909 (60.5)	319 (61.6)	
Late term and postterm (41+ weeks)	214 (15.0)	122 (20.4)		255 (17.0)	81 (15.6)	
Newborn birth weight (grams), No. (%)			<.001			.92
<2500 (underweight)	34 (2.4)	20 (3.4)		39 (2.6)	15 (2.9)	
2500-4000 (normal)	1265 (89.5)	448 (75.8)		1277 (85.5)	436 (85.3)	
>4000 (macrosomic)	115 (8.1)	123 (20.8)		178 (11.9)	60 (11.7)	
Male sex, No. (%)	685 (48.2)	328 (54.8)	.007	753 (50.1)	50.2)	1.00
Dystocia, No. (%)	156 (11.0)	269 (44.9)	<.001	298 (19.8)	127 (24.5)	.03
Cephalopelvic disproportion, No. (%)	8 (0.6)	94 (15.7)	<.001	71 (4.7)	31 (6.0)	.29
Failed induction, No. (%)	0	47 (7.8)	<.001	29 (1.9)	18 (3.5)	.06
Failed vacuum or forceps, No. (%)	0	17 (2.8)	<.001	13 (0.9)	4 (0.8)	1.00
Premature or prolonged rupture of membranes and/or amnionitis, No. (%)	102 (7.2)	52 (8.7)	.27	118 (7.9)	36 (6.9)	.57
Umbilical cord complications, No. (%)	450 (31.6)	116 (19.4)	<.001	431 (28.7)	135 (26.1)	.26
Perineal laceration, 3rd or 4th degree, No. (%)	128 (9.0)	0	<.001	99 (6.6)	29 (5.6)	.47
Cesarean wound complications, No. (%)	0	60 (10.0)	<.001	38 (2.5)	22 (4.2)	.52
Maternal hospital stay > 5 days, No. (%)	4 (0.3)	24 (4.2)	<.001	21 (1.4)	7 (1.4)	1.00

© 2020 Kjerulff et al. *JAMA Netw Open*.

eTable 5. Concurrent-Exposure Covariates by Mode of First Delivery and Subsequent Conception Among Women who Had Unprotected Intercourse Before First Conception or Resulting in no Conception[a, b] (continued)

	**Mode of Delivery**			**Conceived**		
	**Vaginal 1422 (70.4)**	**Cesarean 599 (29.6)**	***P* Value**	**Yes 1503 (74.4)**	**No 518 (25.6)**	***P* Value**
Fetal congenital anomalies, No. (%)	85 (6.0)	46 (7.7)	.17	99 (6.6)	32 (6.2)	.84
5 Minute Apgar < 9, No. (%)	306 (21.6)	157 (26.7)	.02	323 (21.6)	140 (27.6)	.01
Neonatal ICU (NICU) admission, No. (%)	63 (4.4)	36 (6.0)	.14	72 (4.8)	27 (5.2)	.72
Neonatal Hospital length of stay of > 5 days, No. (%)	34 (2.5)	18 (3.3)	.35	39 (2.7)	13 (2.7)	1.00

Abbreviations: ICU, intensive care unit; NICU, neonatal intensive care unit.

[a]Among women who reported having unprotected intercourse before first conception or resulting in no conception and who reported months of unprotected intercourse (n = 2021).

[b]All results reported in this table are from χ^2 analyses.

© 2020 Kjerulff et al. *JAMA Netw Open*.
